# Study of the relation between serum levels of long-acting penicillin and the inflammatory markers: C-reactive protein and interleukin-6 in patients with chronic rheumatic heart disease

**DOI:** 10.1186/s43044-021-00141-0

**Published:** 2021-02-27

**Authors:** Ahmad M. Yousef, Osama A. Rifaie, Mohamed A. Hamza, Sameh A. Amin

**Affiliations:** Cardiology Department, Ain Shams University, Cairo, Egypt

**Keywords:** Long-acting penicillin, C-reactive protein, Interleukin-6, Rheumatic heart disease

## Abstract

**Background:**

There is an evidence of a chronic inflammatory state in patients with chronic rheumatic valvular heart disease (RHD) as shown by high serum levels of high-sensitivity C-reactive protein (hs-CRP) and interleukin-6 (IL6). Despite the efficacy of long-acting penicillin (LAP) in secondary prevention of rheumatic fever, its effect on this inflammatory state is still unknown. So, we sought to study the effect of LAP on the inflammatory markers, CRP and IL-6, in patients with chronic rheumatic heart disease.

**Results:**

Eighty RHD patients coming to our hospital’s outpatient clinic for rheumatic fever secondary prophylaxis by regular administration of LAP were enrolled in the study. Patients were divided into 3 groups: group A, 70 patients with RHD already on prophylactic LAP, group B, 10 patients with RHD who have not yet started prophylactic LAP, and group C, control group of 10 healthy individuals not known to have RHD. Serum levels of LAP, IL-6, and CRP were measured for the three groups.

Group A had significantly lower IL-6 levels than group B (25.22 ± 33.50 vs. 126.1 ± 33.76nng/ml, respectively, *p* < 0.0001). IL-6 levels were significantly lower in control subjects compared to patients in group B (3.600 ± 2.319, 25.22 ± 33.50 ng/ml, respectively, *p* < 0.0001). However, IL-6 levels in the control group were lower but non-significantly different compared to group A. CRP level was lower in group A than group B (8419 ± 4935 vs. 14400 ± 3375 mg/dl, respectively, *p* = 0.0002). CRP levels were significantly lower in control subjects compared to patients in group A and group B. IL-6 values were positively correlated with CRP values (*r* = 0.6387, *p* < 0.0001).

CRP values were negatively correlated with LAP values (*r* = -0.5277, *p* < 0.0001). IL-6 values were negatively correlated with LAP values (*r* = − 0.4401, *p* < 0.0001). There was a highly significant difference between LAP level in compliant and non-compliant patients (1.045 ± 1.270 vs. 0.0785 ± 0.1057 ng/ml, respectively, *p* value < 0.0001).

There was also a highly significant difference between CRP level in compliant and non-compliant patients (7640 ± 4558 vs. 13090 ± 4717 mg/dl, respectively, *p* = 0.005). Moreover, there was a significant difference between IL-6 levels in compliant and non-compliant patients (21.53 ± 32.70 vs. 47.40 ± 30.91 ng/ml, respectively, *p* value 0.03).

**Conclusion:**

Serum LAP has a strong negative correlation with IL-6 and CRP levels. Regular administration of LAP strongly ameliorates the inflammatory state seen in patients with RHD.

## Background

Rheumatic fever is the most important cause of acquired heart disease in children and young adults worldwide. It is an inflammatory reaction that occurs approximately 10 to 21 days after throat infection with virulent strains of group A beta-hemolytic streptococci [1].

C-reactive protein is increased in patients with acute rheumatic fever. High levels of hs-CRP in patients with chronic rheumatic valve disease indicate the persistence of inflammation in the chronic phase [2].

Inflammatory cytokines, such as TNFɑ, IL-8, and IL-6, may play a pathogenic role in rheumatic fever [3].

Single monthly injection of 1,200,000 units of benzathine penicillin confers a high degree of continous protection against group A streptococci and affords reliable means of protecting the patient against recurrences of rheumatic fever [4].

However, the efficacy of long-acting penicillin for secondary prevention of rheumatic fever has not been widely studied; consequently, the relation between serum levels of long-acting penicillin and inflammatory markers C-reactive protein and interleukin-6 is largely unknown.

## Aim of the study

The aim of the study is to study the relation between serum levels of long-acting penicillin and the inflammatory markers C-reactive protein and interleukin-6 in patients with chronic rheumatic heart disease.

## Methods

### Study population

Eighty patients from rheumatic heart disease patients coming to our hospitals’ cardiology outpatient clinic for rheumatic fever prophylaxis by regular long-acting penicillin administration were included in the study. Patients were divided into 2 groups: group A, 70 patients with rheumatic heart disease already on prophylactic long-acting penicillin, and group B, 10 patients with rheumatic heart disease who have not started prophylactic long-acting penicillin yet. Group C is the control group of 10 healthy individuals not known to have rheumatic heart disease or acute inflammatory condition.

### Inclusion criteria

1. All patients with definite diagnosis of chronic rheumatic heart disease, with no age or sex predilection.

### Exclusion criteria

1. Patients with collagen disease and other inflammatory conditions that can cause increased levels of inflammatory mediators.
2. Patients with active infection or acute illness.

All patients were subjected to the following:

1. Detailed medical history was taken from all patients with stress on the following:

- Compliance of patients to the long-acting penicillin regimen and type of this regimen. The patient was considered compliant if he, at least, has taken his last four doses of long-acting penicillin in time
- The risk factors for rheumatic fever, e.g., age, residency, socioeconomic class, family history of rheumatic fever, recurrent streptococcal infection, and documented previous attack of rheumatic fever according to modified Jones criteria
- Past and current medical history regarding inflammatory conditions that can cause increased levels of inflammatory mediators

2. Complete general and local examination has been done for all patients
3. Transthoracic echocardiography done within the last year showing rheumatic valvular heart disease
4. Informed consent from each patient
5. Serum levels of long-acting penicillin, C-reactive protein, and interleukin-6:

- Venous blood samples were drawn under aseptic conditions and centrifuged, and 2 ml serum was collected and stored at minus 20 °C for ELISA at our hospital immunology laboratory. All the serum samples were analyzed for long-acting penicillin, C-reactive protein, and interleukin-6 at our Clinical Pathology Department using ELISA techniques using benzathine benzylpenicillin ELISA kit, high-sensitivity C-reactive protein ELISA kit, and human interleukin-6 ELISA kit.

The current testing methods of CRP and IL-6 including latex agglutination, nephelometry, and radial immunodiffusion (RID) have the general disadvantages of low sensitivity, whereas enzyme-linked immunosorbent assays (ELISA) provide the highest sensitivity and specificity [5, 6].

### Statistical methods

All continuous data were expressed as mean with standard deviation. Comparison between the 3 groups was done using one-way ANOVA method and Kruskal-Wallis tests. Comparison between 2 groups was done using unpaired *t* test or Mann-Whitney tests as appropriate. Linear correlation between 2 variables was done using Spearman correlation coefficient.

*p* value of less than 0.05 was considered significant.

## Results

Basic demographic and clinical data are presented in Table 1.

**Table 1:** Basic demographic and clinical data for all patients and both groups

	All (***n*** = 80)	Group A: on LAP (***n*** = 70)	Group B: not on LAP (***n*** = 10)	***p*** value
**Age (years)**	35.83 ± 11.16	36.17 ± 11.54	33.40 ± 8.044	0.7644
**Gender**				0.333
**Males (%)**	26 (32.5)	22	4	
**Females (%)**	54 (67.5)	48	6	
**Rhythm**				0.3884
**Sinus (%)**	62 (77.5)	56	6	
**AF (%)**	18 (22.5)	14	4	

*LAP* long-acting penicillin, *AF* atrial fibrillation

Valvular affection is shown in Table 2.

**Table 2:** Valvular affection between patients

Valve affection	Number of patients	Percentage (%)
Lone mitral regurge	17 patients	21.25
Lone mitral stenosis	19 patients	23.75
Combined mitral regurge and stenosis	13 patients	16.25
Lone aortic regurge	7 patients	8.75
Lone aortic stenosis	2 patients	2.5
Aortic regurge and mitral regurge	4 patients	5
Aortic regurge and mitral stenosis	3 patients	3.75
Prosthetic valve	12 patients	15
Previous balloon mitral valve	2 patients	2.5
Previous balloon pulmonary valve	1 patient	1.25
Total	80 patients	100

### Long-acting penicillin compliance and regimens

Sixty patients (85.7% of group A) were compliant on long-acting penicillin, and 10 patients (14.3% of group A) were not compliant on long-acting penicillin while 10 patients (100 % of group B) have not started long-acting penicillin.

Thirty patients (48.58% of group A) were on 2-week regimen of long-acting penicillin, eight patients (11.42% of group A) were on 3-week regimen of long-acting penicillin, and 28 patients (40 % of group A) were on 4-week regimen of long-acting penicillin. All the 10 patients (100 % of group B) have not started long-acting penicillin.

### Interleukin-6 and C-reactive protein in patients and control subjects

The mean interleukin-6 value of the control group was significantly lower than that of the patient group (3.600 ± 2.319 vs. 37.83 ± 47.30 ppb, respectively, *p* value < 0.0001).

Surprisingly, unpaired *t* test with Welch’s correction revealed that group A had highly significant lower interleukin-6 levels than group B (25.22 ± 33.50 vs. 126.1 ± 33.76, respectively, *p* value< 0.0001).

**Fig. 1:**
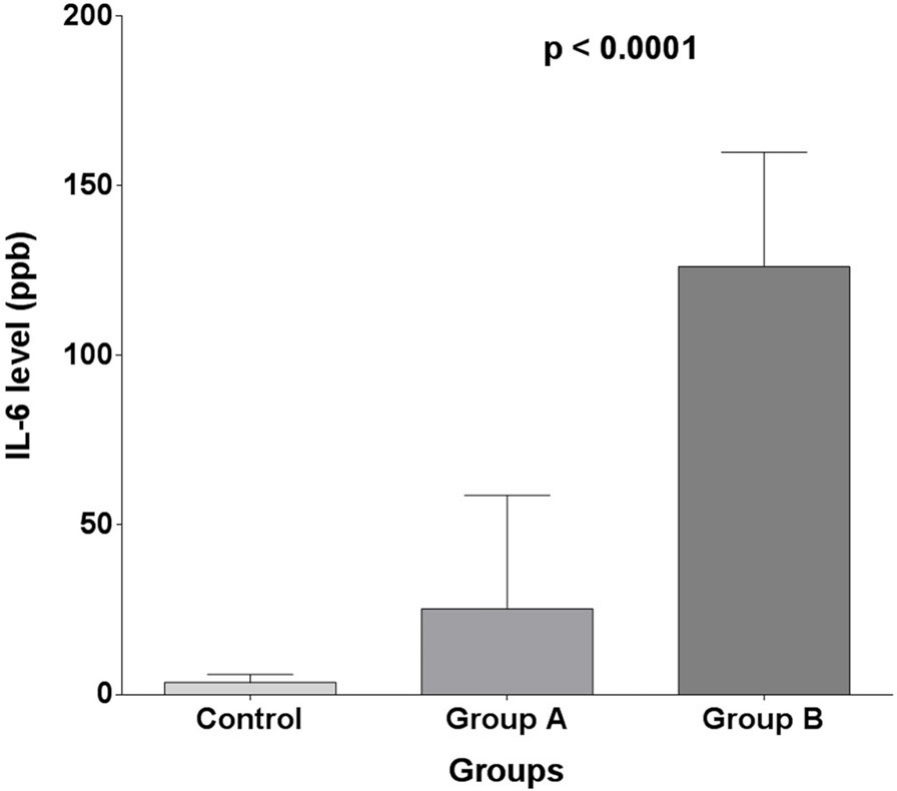
One-way ANOVA test for IL-6 groups

Moreover, both one-way ANOVA test and Kruskal-Wallis test revealed that mean interleukin-6 levels were highly significant different between patients of the control group, group A and group B (3.600 ± 2.319, 25.22 ± 33.50 and 126.1 ± 33.76 ppb, respectively, *p* < 0.0001 (Fig. 1)).

Tukey’s test and Dunn’s multiple comparisons tests also revealed that group A had significantly lower interlukin-6 levels than group B. Interlukin-6 levels were significantly lower in control subjects compared to patients in group B. But interlukin-6 levels in the control group were lower but with non-significant difference compared to patients in group A levels.

The mean C-reactive protein value of the control group was significantly lower than the patient group (2500 ± 3919 vs. 9166 ± 5151 mg/dl, respectively *p* = 0.0003).

Interestingly, unpaired *t* test with Welch’s correction revealed that group A had significantly lower C-reactive protein levels than group B (8419 ± 4935 vs. 14400 ± 3375 respectively, *p* = 0.0002).

**Fig. 2:**
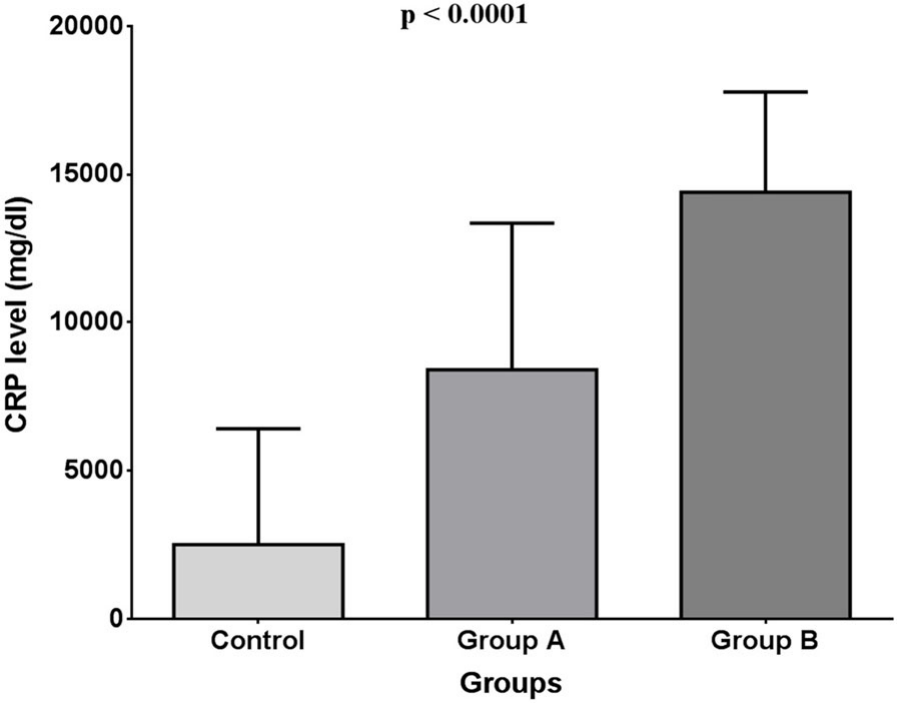
One-way ANOVA test for CRP groups

Moreover, both one-way ANOVA test and Kruskal-Wallis test revealed that mean C-reactive protein levels were significantly different between patients in the control group, group A and group B (2500 ± 3919, 8419 ± 4935 and 14400 ± 3375 respectively, *p* < 0.0001 (Fig. 2)).

Tukey’s test and Dunn’s multiple comparisons tests, interestingly, revealed that C-reactive protein levels were significantly lower in control subjects compared to patients in group A and group B. Group A had significantly lower C-reactive protein levels than group B.

Group A showed positive C-reactive protein values in 35 patients (50% of the patients of group A) while all the 10 patients of group B (100 %) had positive C-reactive protein values, while negative values were detected in 35 patients; all of them were from group A (those on long-acting penicillin).

A comparison between interleukin-6 and C-reactive protein between control subjects and subgroups is shown in Table 3.

**Table 3:** Comparison between interleukin-6 and C-reactive protein between control subjects and subgroups

	Control (group C) (***n*** = 10)	Whole patients (A and B groups) (***n*** = 80)	Group A (on LAP) (***n*** = 70)	Group B (not on LAP) (***n*** = 10)	***p*** value
		mean ± SD	mean ± SD	mean ± SD	
**CRP (mg/dL)**	2500 ± 3919	9166 ± 5151	8419 ± 4935	14400 ± 3375	< 0.0001
**IL-6 (ppb)**	3.600 ± 2.319	37.83 ± 47.30	25.22 ± 33.50	126.1 ± 33.76	< 0.0001

*LAP* long-acting penicillin, *CRP* C-reactive protein, *IL-6* interleukin-6, *SD* standard deviation

### Correlations

#### Interlukin-6 and C-reactive protein correlation

**Fig. 3:**
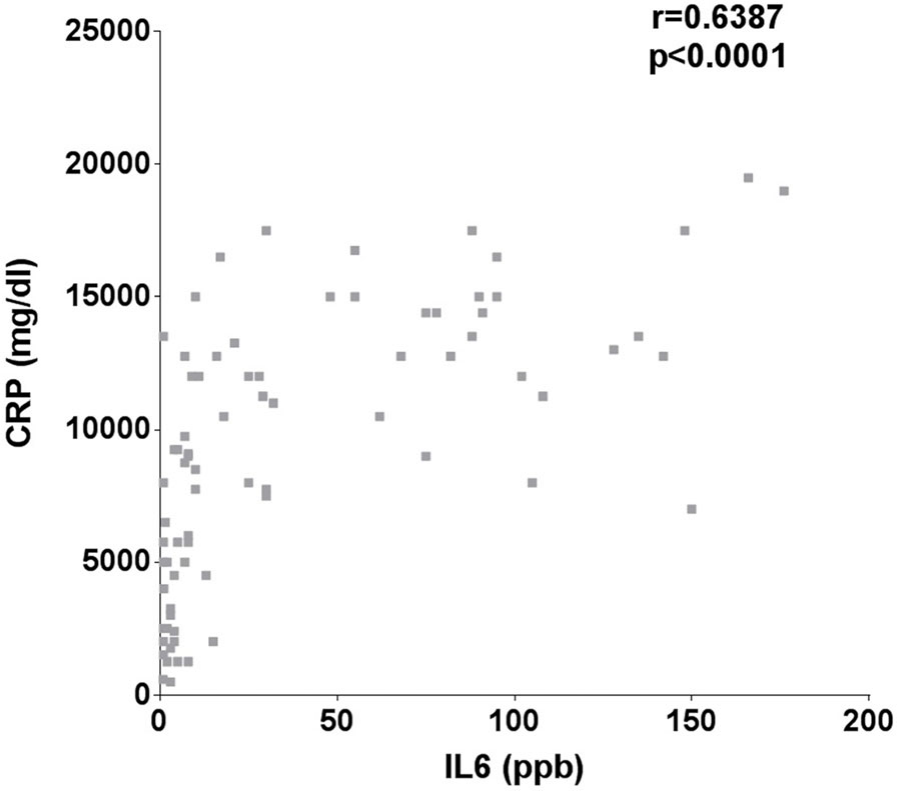
Correlation between IL-6 and CRP

All interleukin-6 values were strongly correlated with C-reactive protein values (*r* = 0.6387, *p* < 0.0001 (Fig. 3)).

#### Correlation between long-acting penicillin and C-reactive protein

**Fig. 4:**
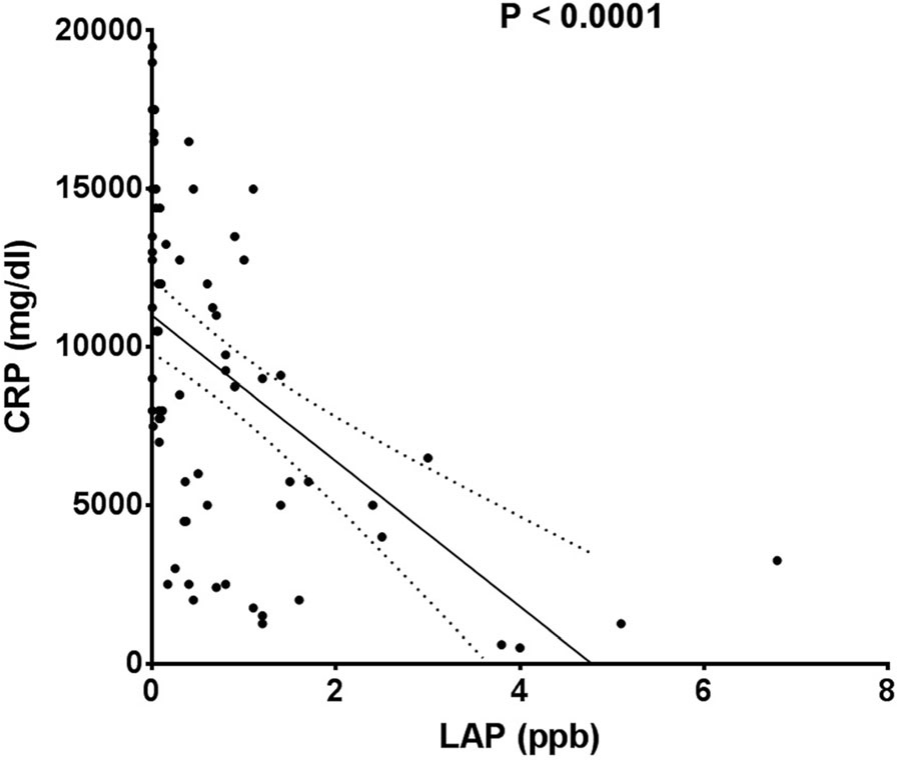
Linear regression between LAP and CRP

C-reactive protein and long-acting penicillin levels had a significant negative correlation (*r* = − 0.5277, *p* < 0.0001 (Fig. 4)).

#### Correlation between long-acting penicillin and interleukin-6

**Fig. 5:**
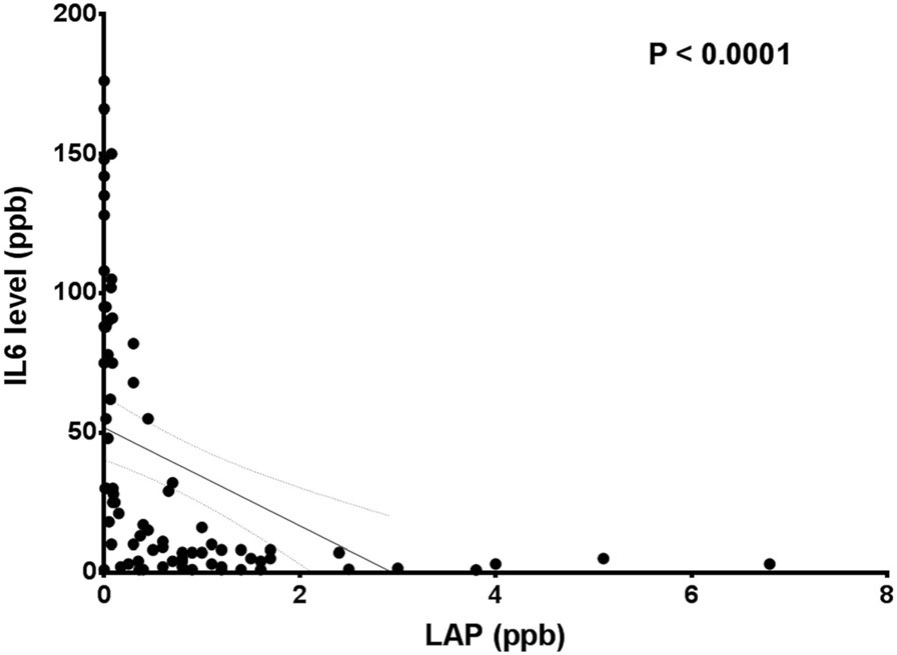
Linear regression between LAP and IL-6

Interleukin-6 level had significantly negative correlation with long-acting penicillin level (*r* =− 0.4401, *p* < 0.0001 (Fig. 5)).

### Compliance to long-acting penicillin and levels of long-acting penicillin, interleukin-6, and C-reactive protein

The patient was considered compliant if he, at least, has taken his last four doses of long-acting penicillin in time.

**Fig. 6:**
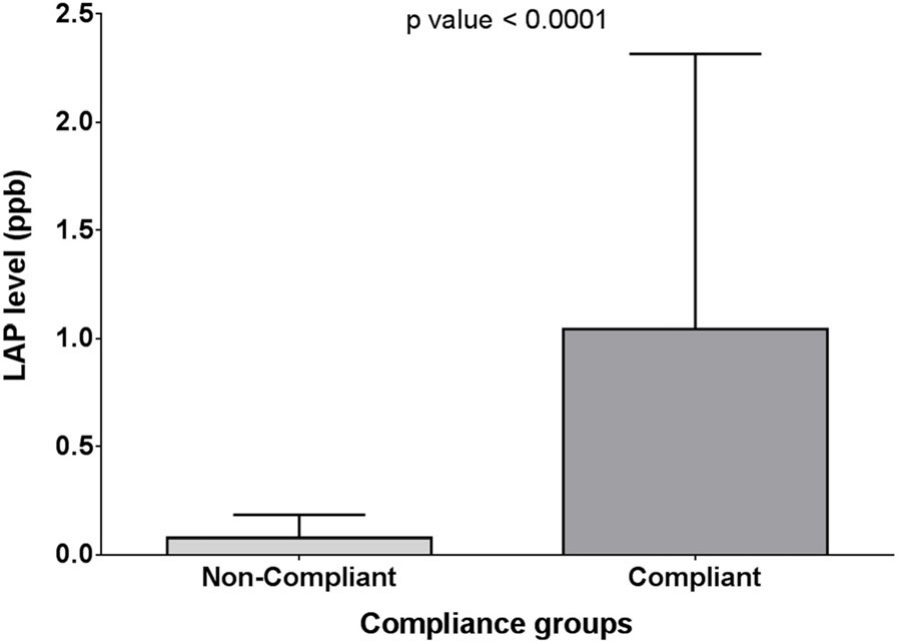
Mean and SD of LAP compliance groups

As regards compliance, there was a highly significant difference between long-acting penicillin level in compliant and non-compliant patients (1.045 ± 1.270 vs. 0.0785 ± 0.1057, respectively with *p* < 0.0001 (Fig. 6)).

**Fig. 7:**
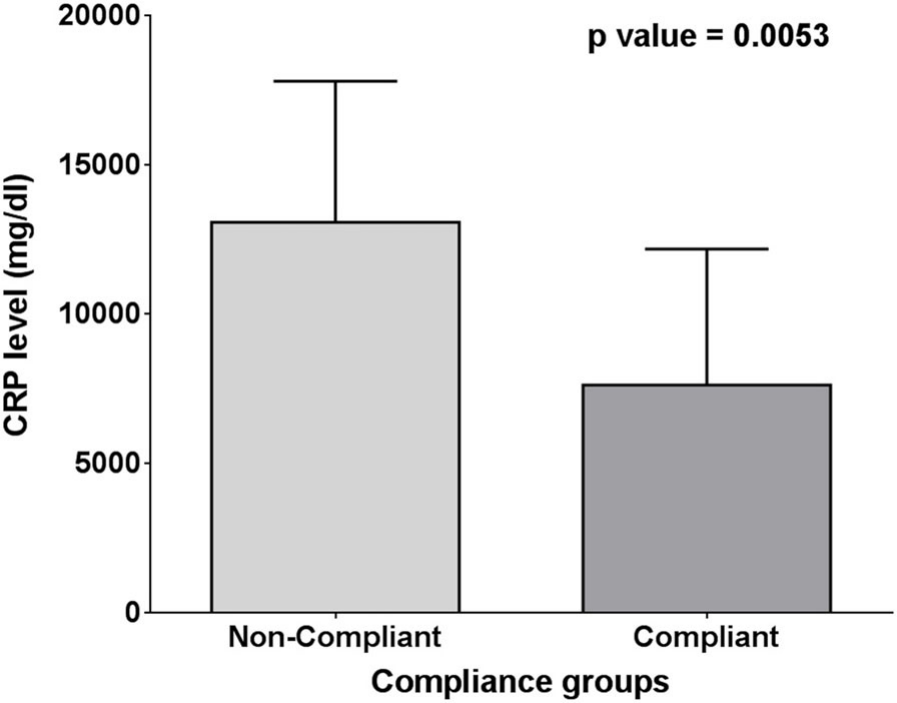
Mean and SD of CRP compliance groups

There was also a significant difference between C-reactive protein level in compliant and non-compliant patients (7640 ± 4558 vs. 13090 ± 4717, respectively and *p* = 0.0053 (Fig. 7)).

**Fig. 8:**
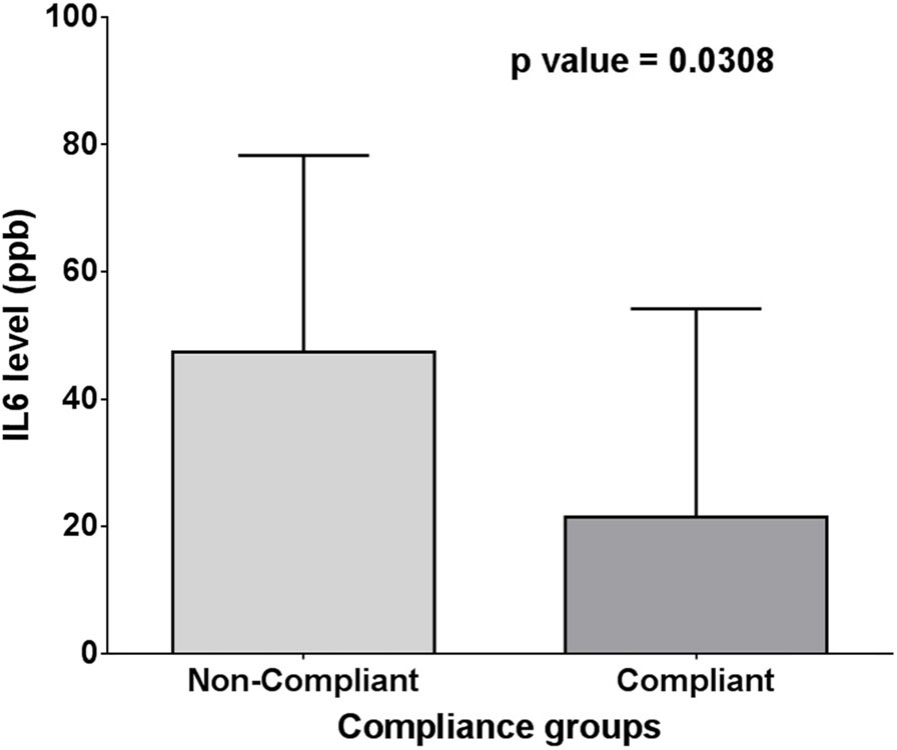
Mean and SD of IL-6 compliance groups

Moreover, there was a significant difference between interleukin-6 levels in compliant and non-compliant patients (21.53 ± 32.70 vs. 47.40 ± 30.91, respectively and *p* = 0.0308) (Fig. 8)).

### Long-acting penicillin regimen and levels of long-acting penicillin, interleukin-6, and C-reactive protein

**Fig. 9:**
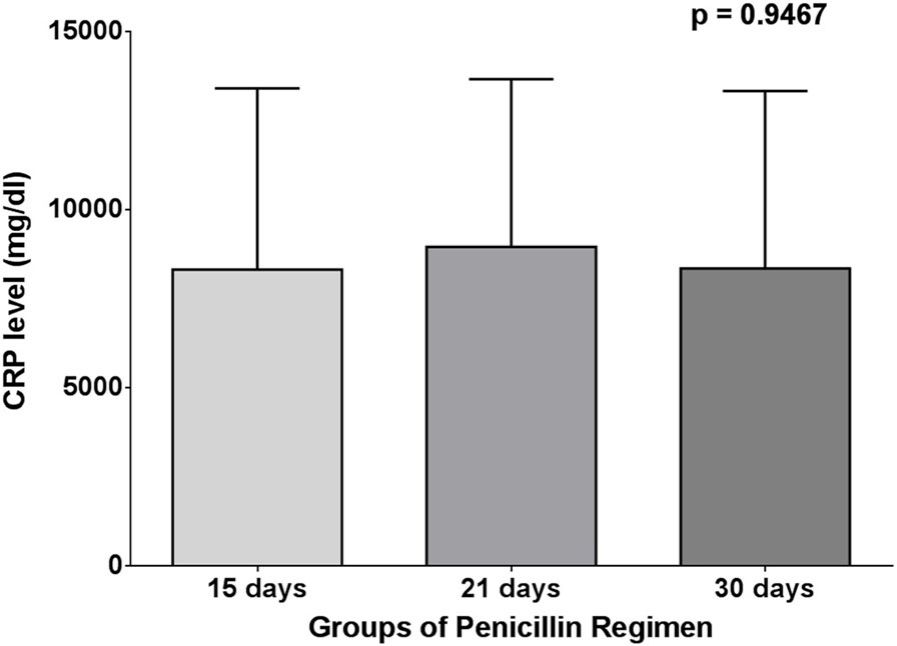
One-way ANOVA test for CRP between penicillin and regimen groups

**Fig. 10:**
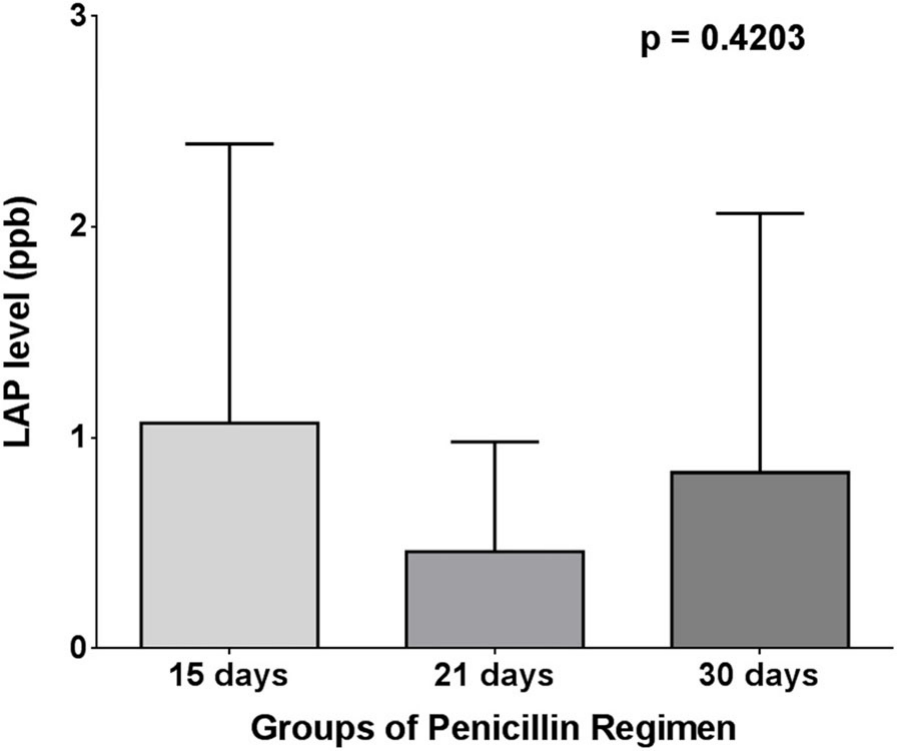
One-way ANOVA test for LAP between penicillin and regimen groups

**Fig. 11:**
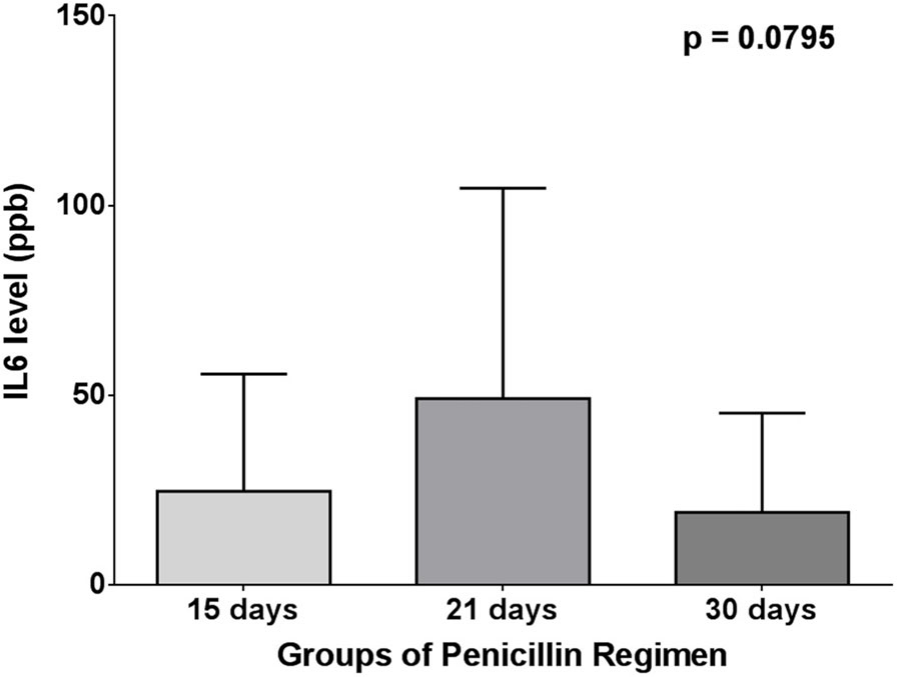
One-way ANOVA test for IL-6 between penicillin and regimen groups

Both one-way ANOVA test and Kruskal-Wallis test, surprisingly, showed that there was no significant difference in long-acting penicillin, C-reactive protein, and interleukin-6 levels (*p* = 0.4203, 0.9467 and 0.0795, respectively) between 15-, 21-, and 30-day regimens of penicillin (Figs. 9, 10 and 11).

Table 4 shows the correlation between long-acting penicillin regimen and levels of long-acting penicillin, interleukin-6, and C-reactive protein.

**Table 4:** Correlation between long-acting penicillin regimen and levels of long-acting penicillin, interleukin-6, and C-reactive protein

	LAP every 15 days (***n*** = 34) (48.58%)		LAP every 21 days (***n*** = 8) (11.42%)		LAP every 30 days (***n*** = 28) (40 %)		***p*** value
	Mean	SD	Mean	SD	Mean	SD	
**LAP (ppb)**	1.070	1.326	0.4609	0.5199	0.8364	1.229	0.4203
**IL-6 (ppb)**	24.65	30.99	49.13	55.50	19.09	26.19	0.0795
**CRP (mg/dL)**	8335	5085	8969	4693	8363	4983	0.9467

*LAP* long-acting penicillin, *CRP* C-reactive protein, *IL-6* interleukin-6, *SD* standard deviation

### Rhythm and levels of long-acting penicillin, interleukin-6, and C-reactive protein

Surprisingly, there was no significant difference in long-acting penicillin, C-reactive protein, and interleukin-6 levels (*p* = 0.3421, 0.3050, and 0.6758, respectively) between sinus patients and atrial fibrillation patients.

Table 5 shows the correlation between rhythm and levels of long-acting penicillin, interleukin-6, and C-reactive protein.

**Table 5:** Relation between rhythm and LAP, IL-6, and CRP

	Sinus rhythm (***n*** = 56)		AF rhythm (***n*** = 14)		***p*** value
	Mean	SD	Mean	SD	
**LAP (ppb)**	0.9697	1.268	0.6568	1.028	0.3421
**IL-6 (ppb)**	25.99	34.66	22.14	29.33	0.6758
**CRP (mg/dL)**	8068	4703	9821	5748	0.3050

*LAP* long-acting penicillin, *CRP* C-reactive protein, *IL-6* interleukin-6, *AF* atrial fibrillation, *SD* standard deviation

## Discussion

Several studies concluded that following an acute attack of rheumatic fever, there was a continuous chronic inflammation of cardiac valves proportionate to the degree of valve affection [7–9]. In fact, a positive correlation was found between c-reactive protein level and severity of valvular dysfunction [2, 7].

Some studies such as Yegin O. et al. [3] and Guilherme L. et al. [10] showed that interleukin-6 plays a pathogenic role in rheumatic activity.

Historically, there are three main studies comparing penicillin to no preventive treatment; one of them showed a 55% reduction in the recurrence of rheumatic fever [11], while the other two revealed a non-significant trend in favor of penicillin [12, 13].

There are three main principal findings of our study. Firstly, there is a strong negative correlation between long-acting penicillin serum level and the inflammatory markers. This finding adds to the evidence that long-acting penicillin is effective in secondary prevention of rheumatic fever. The second finding is as follows: compliance to long-acting penicillin regimen has an important effect on the prophylaxis of rheumatic heart disease. The third finding is as follows: there is a strong correlation between C-reactive protein and interleukin-6 levels in chronic rheumatic heart disease patients especially in patients not taking long-acting penicillin suggesting that there is a continuous chronic inflammation in rheumatic heart disease patients.

In our study, plasma C-reactive protein level and interleukin-6 level were significantly correlated to each other, and this result can be explained by the hypotheses that following acute RF attack, there will be a continuous chronic inflammation of cardiac valves and patients with more intense inflammatory reaction have a more rapid progression of valvular dysfunction [7–9] and that C-reactive protein and interleukin-6 levels are correlated to each other and to the inflammatory process in chronic rheumatic heart disease patients [3].

C-reactive protein and interleukin-6 mean levels were significantly higher in patients with rheumatic heart disease not taking long-acting penicillin compared to patients with rheumatic heart disease taking long-acting penicillin and to the control group. Those findings may support the hypothesis that long-acting penicillin is a strong secondary preventive method of rheumatic heart disease activity [11].

Also, we detected a significant negative correlation between long-acting penicillin levels and both C-reactive protein and interleukin-6 levels. To our knowledge, this is the first study to prove that long-acting penicillin objectively ameliorates the chronic inflammatory state in patients with chronic rheumatic valvular heart disease. This finding can be a further clue in the favor of the recommendation that long-acting penicillin should be administered indefinitely in these patients.

Another finding in the same context was that the mean levels of long-acting penicillin, C-reactive protein, and interleukin-6 in compliant patients to their long-acting penicillin regimen were significantly higher than the mean levels in non-compliant patients. This finding can also be a further clue in the context of the hypothesis that long-acting penicillin is a strong secondary preventive method of rheumatic heart disease activity.

In our study, the mean levels of long-acting penicillin, C-reactive protein, and interleukin-6 in each regimen of long-acting penicillin (every 15 days, every 21 days, and every 30 days) were not significantly different. This finding is different from findings of Kassem A. S. et al. [14] that concluded a superiority of the 2-weekly schedule in the adequate control of RF recurrences.

Our study depends only on the serum levels of long-acting penicillin, C-reactive protein, and interleukin-6. There are many other factors that may affect the regimen results that we did not include in our study like age difference, weight difference, and difference in compliance definition.

We observed that there is no correlation between cardiac rhythm and C-reactive protein, interleukin-6, or long-acting penicillin levels. This result conflicts with the hypothesis that rheumatic heart disease patients with higher levels of C-reactive protein are more prone to AF as noticed by Attar A. et al. [7], Ucer E. et al. [15], and Selcuk M. T. et al. [16]. This may be due to the different weights of the patients and the different echocardiographic valvular lesions in our study instead of the dominance of mitral stenosis in other studies [11].

In fact, it is difficult to know whether or not this chronic inflammatory state in these patients is a result of ongoing streptococcal infection which triggers the auto immune disease. However, interleukin-6 was shown to be involved in the pathogenesis of rheumatic fever. Thus, the rise of interleukin-6 may denote an active chronic rheumatic fever. This finding may further suggest that addition of anti-inflammatory drugs as corticosteroids and non-steroidal anti-inflammatory agents targeting the inflammatory mediators might help to control the progress of rheumatic heart disease.

## Limitations of the study

Our study has the following limitations: first is the small number of patients coming from a single medical center (Ain Shams University Hospitals).Secondly, we did not study the cause and effect relationship between long-acting penicillin, C-reactive protein, interleukin-6 serum levels, and valvular damage in RHD. Thirdly, no comparison was done between the serum level of C-reactive protein and interleukin-6 before and after administration of long-acting penicillin in the same patient.

## Conclusions

Serum long-acting penicillin has a strong negative correlation with interleukin-6 and C-reactive protein levels. This is probably the first study to show that regular administration of long-acting penicillin strongly ameliorates the chronic inflammatory state that accompanies chronic rheumatic heart disease.

## Data Availability

The datasets used and/or analyzed during the current study are available from the corresponding author on reasonable request.

## Abbreviations

CRP: C-reactive protein
IL-6: Interleukin-6
LAP: Long-acting penicillin
ppb: Part per billion
RF: Rheumatic fever
RHD: Rheumatic heart disease

## References

[1] Manyemba J, Mayosi BM (2002). Penicillin for secondary prevention of rheumatic fever. Cochrane Database Syst Rev.

[2] Golbasi Z, Ucar O, Keles T, Sahin A, Cagli K, Camsari A (2002). Increased levels of high sensitive C-reactive protein in patients with chronic rheumatic valve disease: evidence of ongoing inflammation. Eur J Heart Fail.

[3] Yegin O, Coskun M, Ertug H (1997). Cytokines in acute rheumatic fever. Eur J Pediatr.

[4] Stollerman GH, Rusoff JH, Hirschfeld I (1955). Prophylaxis against group A streptococci in rheumatic fever; the use of single monthly injections of benzathine penicillin G. N Engl J Med.

[5] Van Leeuwen M, Van Rijswijk MH (1994). Acute phase proteins in monitoring inflammatory disorders. J Bailliere’s Clinical Rheumatology.

[6] Roberts WL (2000). Evaluation of four automated high-sensitivity C-reactive protein methods: implications for clinical and epidemiological applications. J Clinical Chemistry.

[7] Attar A, Marzban P, Moaref A, Aghasadeghi K (2018). The association of plasma high-sensitivity C-reactive protein level with rheumatic heart disease: the possible role of inflammation. Indian Heart J.

[8] Iman A, Akbar MA, Mohsen KM, Ali F, Armin A, Sajjad A (2013). Comparison of intradermal injection of autologous epidermal cell suspension vs. spraying of these cells on dermabraded surface of skin of patients with post-burn hypopigmentation. Indian J Dermatol.

[9] Attar A, Ghalyanchi Langeroudi A, Vassaghi A, Ahrari I, Maharlooei MK, Monabati A (2013). Role of CD271 enrichment in the isolation of mesenchymal stromal cells from umbilical cord blood. Cell Biol Int.

[10] Guilherme L, Cury P, Demarchi LM, Coelho V, Abel L, Lopez AP (2004). Rheumatic heart disease: proinflammatory cytokines play a role in the progression and maintenance of valvular lesions. Am J Pathol.

[11] Padmavati S, Sharma KB, Jayaram O (1973). Epidemiology and prophylaxis of rheumatic fever in Delhi--a five year follow-up. Singap Med J.

[12] Feinstein AR (1966). The natural histories of acute rheumatic fever. Bull Rheum Dis.

[13] Kohn KH, Milzer A, Maclean H (1953). Prophylaxis of recurrences of rheumatic fever with penicillin given orally; final report of a five year study. J Am Med Assoc.

[14] Kassem AS, Madkour AA, Massoud BZ, Zaher SR (1992). Benzathine penicillin G for rheumatic fever prophylaxis: 2-weekly versus 4-weekly regimens. Indian J Pediatr.

[15] Ucer E, Gungor B, Erdinler IC, Akyol A, Alper AT, Eksik A (2008). High sensitivity CRP levels predict atrial tachyarrhythmias in rheumatic mitral stenosis. Ann Noninvasive Electrocardiol.

[16] Selcuk MT, Selcuk H, Maden O, Temizhan A, Aksu T, Dogan M (2007). Relationship between inflammation and atrial fibrillation in patients with isolated rheumatic mitral stenosis. J Heart Valve Dis.

